# Nordihydroguaiaretic acid reduces secondary organ injury in septic rats after cecal ligation and puncture

**DOI:** 10.1371/journal.pone.0237613

**Published:** 2020-08-13

**Authors:** Michael E. Zubrow, Susan S. Margulies, Nadir Yehya

**Affiliations:** 1 Division of Pediatric Critical Care Medicine, Barbara Bush Children’s Hospital at Maine Medical Center, Portland, ME, United States of America; 2 Wallace H. Coulter Department of Biomedical Engineering, Georgia Tech College of Engineering, Emory University School of Medicine, Atlanta, GA, United States of America; 3 Department of Anesthesiology and Critical Care Medicine, Children’s Hospital of Philadelphia and University of Pennsylvania, Philadelphia, PA, United States of America; University of Alabama at Birmingham, UNITED STATES

## Abstract

**Background:**

Nordihydroguaiaretic acid (NDGA) is a plant extract that has been shown to act as a free radical scavenger and pluripotent inhibitor of pro-inflammatory cytokines, two major cellular processes involved in the pathophysiology of sepsis. We investigated whether NDGA would improve markers of organ injury as well as survival in a rodent model of sepsis.

**Methods:**

Abdominal sepsis was induced by cecal ligation and double puncture (CLP) in male Sprague-Dawley rats. NDGA was administered either at the time of injury (pre-) or 6 hours later (post-treatment). A sham surgery group and a vehicle only group were also followed as controls. Blood and lung tissue were collected 24 h after CLP. Lung tissue was used for histopathologic analysis and to measure pulmonary edema. Arterial oxygenation was measured directly to generate PaO2/FiO2, and markers of renal injury (blood urea nitrogen), liver injury (alanine aminotransferase), and tissue hypoxia (lactate) were measured. In a separate set of animals consisting of the same treatment groups, animals were followed for up to 36 hours for survival.

**Results:**

NDGA pre-treatment resulted in improved oxygenation, less lung edema, lower lactate, lower BUN, and reduced histologic lung injury. NDGA post-treatment resulted in less lung edema, lower lactate, lower BUN, and less histologic lung injury, but did not significantly change oxygenation. None of the NDGA treatment groups statistically affected ALT or creatinine. NDGA pre-treatment showed improved survival compared with control CLP animals at 36 hours, while post-treatment did not.

**Conclusions:**

NDGA represents a novel pleiotropic anti-inflammatory agent with potential clinical utility for modulation of organ injury secondary to sepsis.

## Introduction

Sepsis is the leading cause of death worldwide among pediatric patients and accounts for over 720,000 hospitalizations in the United States alone annually [1,2]. Sepsis occurs in response to an infectious trigger, leading to systemic release of inflammatory mediators such as IL-1, TNF-alpha, and IL-8 [3,4]. This in turns leads to a positive feedback loop in which dysregulated and prolonged neutrophil recruitment causes release of toxic oxygen species and proteolytic enzymes leading to capillary dysfunction, disordered coagulation, bioenergetic failure increased lipid peroxidation, and secondary organ injury [5–9]. Multiple organ dysfunction in sepsis is associated with an increase in mortality [1], and despite advances in supportive treatment strategies, it remains a disease process with high mortality in both adults and children [10,11]. At present, pharmacologic therapies to modify the immune response or ameliorate reactive oxygen species remain unproven.

Nordihydroguaiaretic acid (NDGA), a plant product of the creosote bush that grows in the southwestern United States, has been shown to inhibit multiple cellular processes involved in this inflammatory process, including pathways of inflammation such as IL-1 and IL-8 [12,13], leukocyte chemotaxis [14], and TNF-alpha induced signal transduction [15], primarily through its action as a lipoxygenase inhibitor. It also has potent anti-oxidant properties and serves as a modulator of lipid peroxidation at the mitochondrial level [16], with more efficient free-radical scavenging properties in vitro than glutathione and N-acetylcysteine [17]. Due to its unique pluripotency in affecting many of the overlapping inflammatory initiators as well as down-stream injurious effectors in the pathophysiology of sepsis, NDGA represents a novel treatment modality. We tested the hypothesis that NDGA given either prior to induction of sepsis via cecal ligation and double puncture (CLP) or 6 hours after would reduce markers of organ injury in a rat model of abdominal sepsis.

## Materials and methods

### Animal care

Adult (8–10 week) male Sprague-Dawley rats weighing 250-350g (Charles River, Boston, MA) were used in all experiments. Rats were given a 1-week acclimation period in a temperature controlled, 12-hour light:dark cycle facility and were fed standard rodent chow and water *ad libitum* both before and during the experimental protocol. All animal protocols were approved by the Institutional Animal Care and Use Committee of the University of Pennsylvania and all care and handling of animals was in accordance with the criteria outlined by National Institutes of Health guidelines.

### Experimental design

Animals were randomly divided in to 4 groups (5 animals per group for serologic studies, 8 animals per group for survival studies): (1) sham surgery + ip NDGA, (2) CLP + dimethylsulfoxide vehicle only, (3) CLP + pre-treatment (concurrent with surgery) with ip NDGA, (4) CLP + treatment with ip NDGA 6 hours after CLP (post-treatment).

Prior to surgery, NDGA (Sigma Chemical Company, St. Louis, MO) was dissolved in dimethylsulfoxide. For experimental groups the receiving drug, NDGA was administered at a dose of 20 mg/kg, as previous studies have shown an LD50 of 75/mg/dose [18]. Anesthesia was induced and maintained with isoflurane (3–4% for induction, 2–3% for maintenance) and sepsis was induced via CLP. A 1.5 cm incision was made in the midline of the ventral aspect of the abdomen and the cecum was exposed, isolated, and ligated with 4–0 silk suture just distal to the ileocecal valve to avoid intestinal obstruction. The cecum was subsequently punctured twice with an 18-gauge needle and compressed gently to extrude feces through the puncture site. Cecum was subsequently returned to the abdominal cavity without removing adherent feces and the abdomen was closed in two layers. Sham-operated rats underwent laparotomy with identical incision and cecal isolation to above, but without ligation or puncture. At the end of surgical intervention, rats were fluid resuscitated with 10mL subcutaneous saline and given 0.1 mg/kg buprenorphine for analgesia. Rats received additional boluses of 10 mL subcutaneous saline and 0.1 mg/kg buprenorphine every 12 hours post-surgical intervention. Animals were not given antibiotics. All experimental staff were trained in procedural sedation, animal monitoring, delivery of fluids and analgesia, and performing of the procedure by members of the University of Pennsylvania Institutional Care and Use Committee.

Animals were sacrificed under isoflurane anesthesia 24 hours after surgery to collect specimens. Blood was collected by left ventricular puncture for assessment of arterial blood. Tracheostomy was performed and right lower lobe of the lung clamped and excised for wet/dry measurements. Remainder of the lung was inflated and fixed in 10% formalin solution for histologic examination. A separate set of animals was followed for up to 36 hours to assess survival.

For all animal experiments, animals were assessed every 6 hours. To minimize suffering to the experimental animals, if there was evidence of any of the following on clinical examination, animals were immediately euthanized under isoflurane anesthetic: impaired mobility, evidence of muscle atrophy or loss of body conditioning, inability to maintain upright position, labored breathing or cyanosis, prolonged decreased food or water intake, inability to eat or drink, prolonged diarrhea, bleeding from any orifice, repeated self-mutilation, or unconsciousness.

### Blood gas measurements

Whole blood was placed into heparinized syringe and placed on ice after specimen obtained from rats. Specimens were then run immediately to obtain PaO2 and lactate in a blood gas analyzer (Nova Biomedical, Waltham, MA), with the remainder stored at -80C.

### Pulmonary edema

We estimated lung edema by lung wet/dry ratio. The right lower lobe of the lung was separated as above, weighed fresh, and then dried in an oven at 80 degrees C for 72 hours, after which it was re-weighed to determine its dry weight.

### Pathology analysis

Formalin-fixed lungs were handled by the Children’s Hospital of Philadelphia Pathology Core Laboratory, where they were processed to paraffin and sectioned to deliver three slides per lung (upper, middle, and lower sections of bilateral lungs). Slides were stained with hematoxylin and eosin and scored using a previously described lung injury score [19] that assigns weighted scores based on the presence of neutrophilic infiltration, hyaline membrane formation, septal thickening, and airspace proteinaceous debris, with scores ranging from 0 to 100. Five high powered fields were scored on each lung at each level, yielding 30 fields analyzed for each animal. Slides were scored blindly without animal identifying information.

### Serum measures of renal and hepatic inflammation

As measures of renal and hepatic inflammation 24 hours after CLP, serum blood urea nitrogen (BUN), creatinine (Cr), aspartate aminotransferase (AST), and alanine aminotransferase (ALT) were measured by a commercial laboratory (Texas A&M, College Station, TX)

### Serum measure of inflammation

As a measure of inflammatory state, serum IL-1b measurement was performed by enzyme-linked immunosorbent assay (ELISA) kit (Ray Biotech, Peachtree Corners, GA). Procedures were carried out according to the instructions provided by the manufacturer.

### Statistical analysis

Data are presented as mean +/- standard error of the mean unless otherwise noted. Survival statistics were compared with an overall log-rank test testing for differences between Kaplan-Meier survival curves. Continuous data were analyzed by two-way analysis of variance with Dunnett’s post-hoc test. In all cases, a p < 0.05 was used to indicate significance.

## Results

### Lung injury—oxygenation

**Fig 1:**
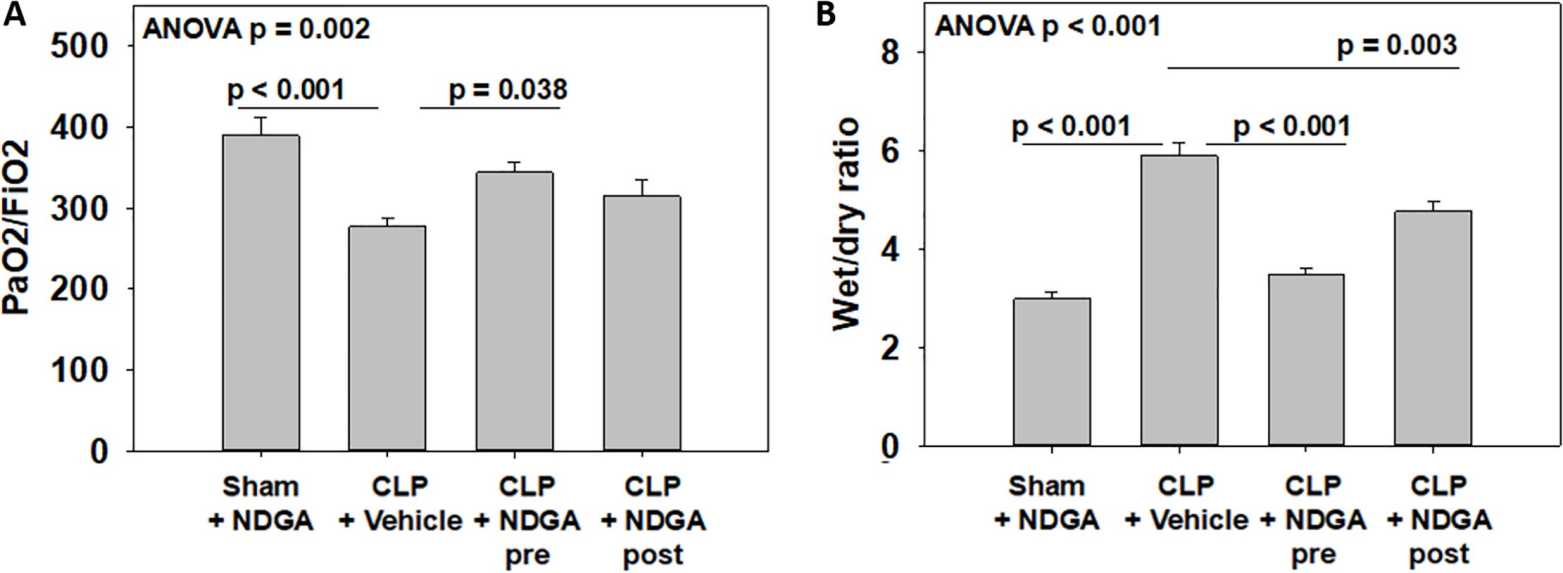
Quantification of lung injury. (A) Quantification of functional lung injury as assessed by oxygenation in the form of PaO2/FiO2 ratio. A significant improvement in oxygenation is seen when NDGA is given prior to CLP (n = 5 in each group). (B) Pulmonary Edema as measured by lung wet to dry ratio. Significant improvements were seen among groups given NDGA both before and 6h after CLP when compared with CLP animals (n = 5 in each group).

Fig 1A shows the PaO2/FIO2 ratios as a marker of adequacy of oxygenation in each of the experimental groups. The CLP-vehicle group had a statistically significant worsening of P/F ratio (276.8) compared with both the sham (389.6, p<0.001) and CLP-NDGA-pre groups (344.0, p = 0.038), but there was no difference when compared with the CLP-NDGA-post group (314.4, p = 0.323).

### Lung injury—pulmonary edema

The CLP-Vehicle group led to significantly more pulmonary edema as compared with a sham surgery when comparing wet to dry ratio (Fig 1B). Both pre- and post-treatment with NDGA led to significantly less pulmonary edema when compared with the CLP-vehicle group (p<0.001 and p = 0.003).

### Lung injury—histology

**Fig 2:**
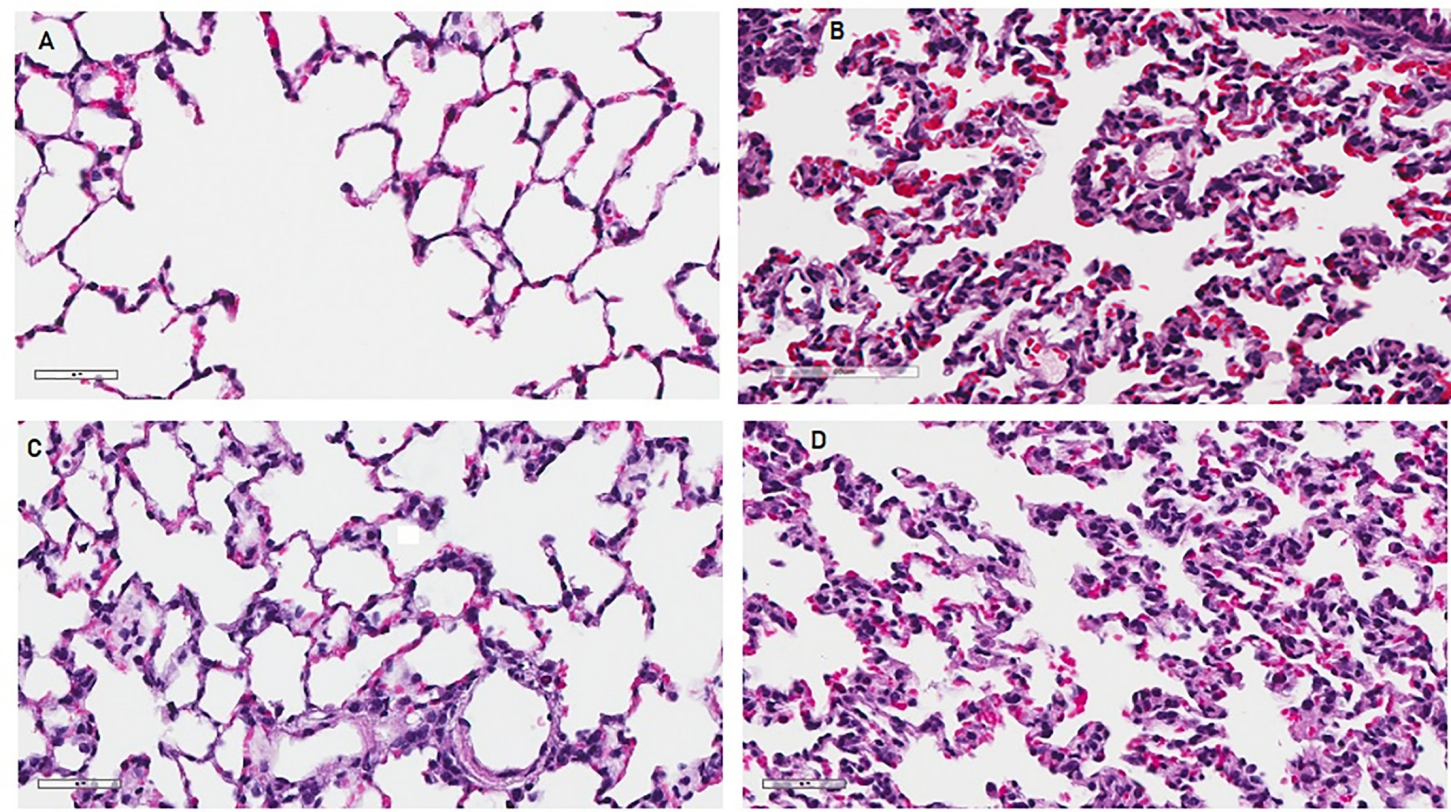
Representative histologic sections of lung taken from rats among various treatment groups. (A) Sham + NDGA surgery group showing normal lung architecture without neutrophilic infiltrate, septal thickening, or hyaline membranes; (B) CLP + vehicle group, showing pronounced septal thickening with significant deposition of hyaline membranes and neutrophilic infiltrate; (C) CLP + NDGA pre-treatment group, showing mild septal thickening, but reduced neutrophilic infiltrate and hyaline deposition; (D) CLP + NDGA post treatment group showing mild to moderate septal thickening with some hyaline membrane deposition and mild neutrophilic infiltrate.

**Fig 3:**
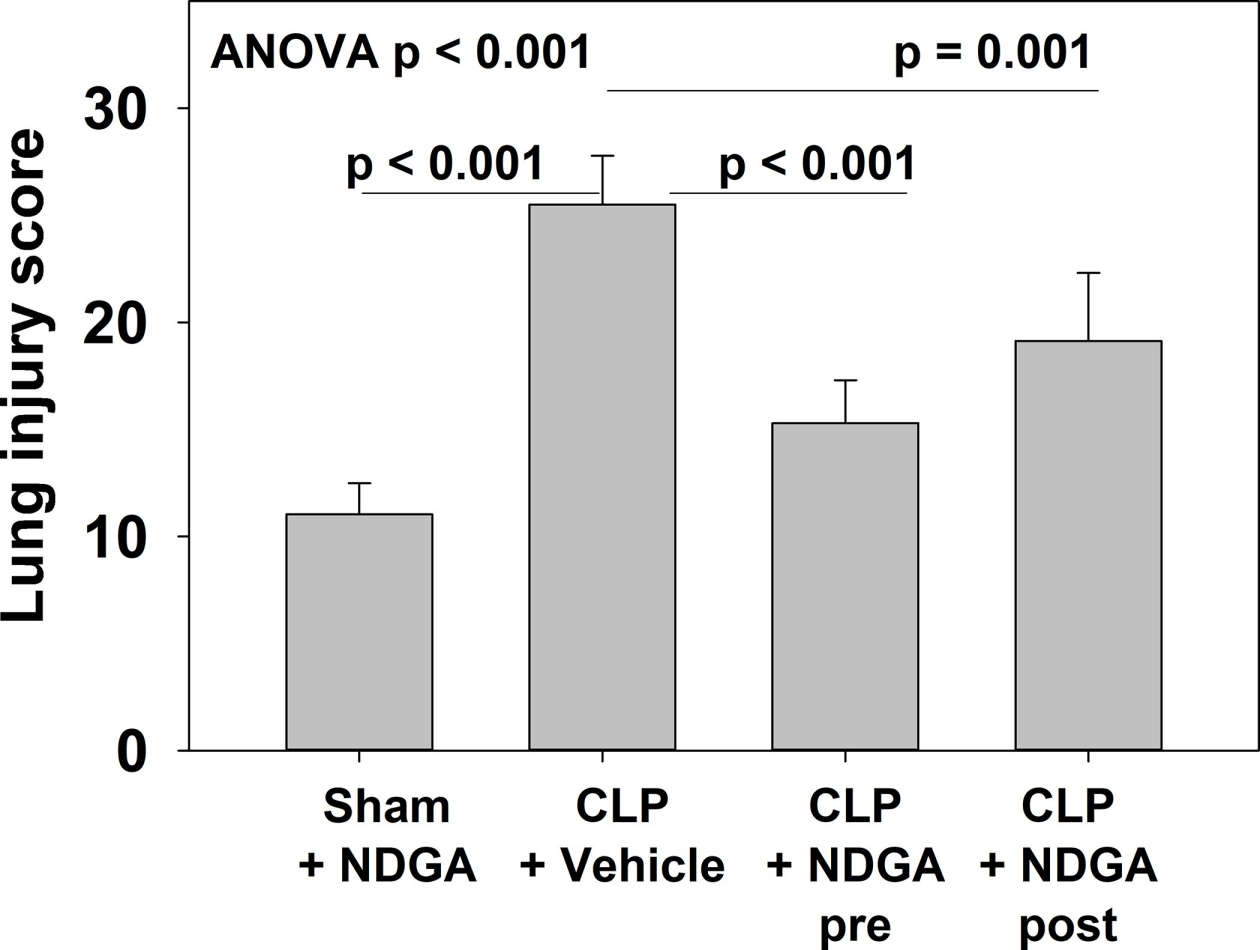
Quantification of the degree of lung injury as measured by lung injury score. A significantly lower degree of lung injury score is seen among groups given NDGA both before and 6h after CLP when compared with CLP animals (n = 5 in each group).

Histologically, CLP-vehicle group animals had significant evidence of lung injury and pathologic change, as seen with neutrophil infiltration, thickening of alveolar walls, and proteinaceous debris (Fig 2). When comparing these groups with a lung injury score to quantitate these changes, CLP-vehicle group animals showed significantly more injury than sham animals, CLP-NDGA-pre-treated animals, and CLP-NDGA-post treated animals (Fig 3). The sham animals had lower lung injury scores (11.0) than the CLP-vehicle group (25.5, p<0.001). Both the NDGA-pre (15.3, p<0.001) and NDGA-post treatment (19.1, p = 0.001) groups also had significantly lower injury scores than the CLP-vehicle group.

### Metabolic injury

**Fig 4:**
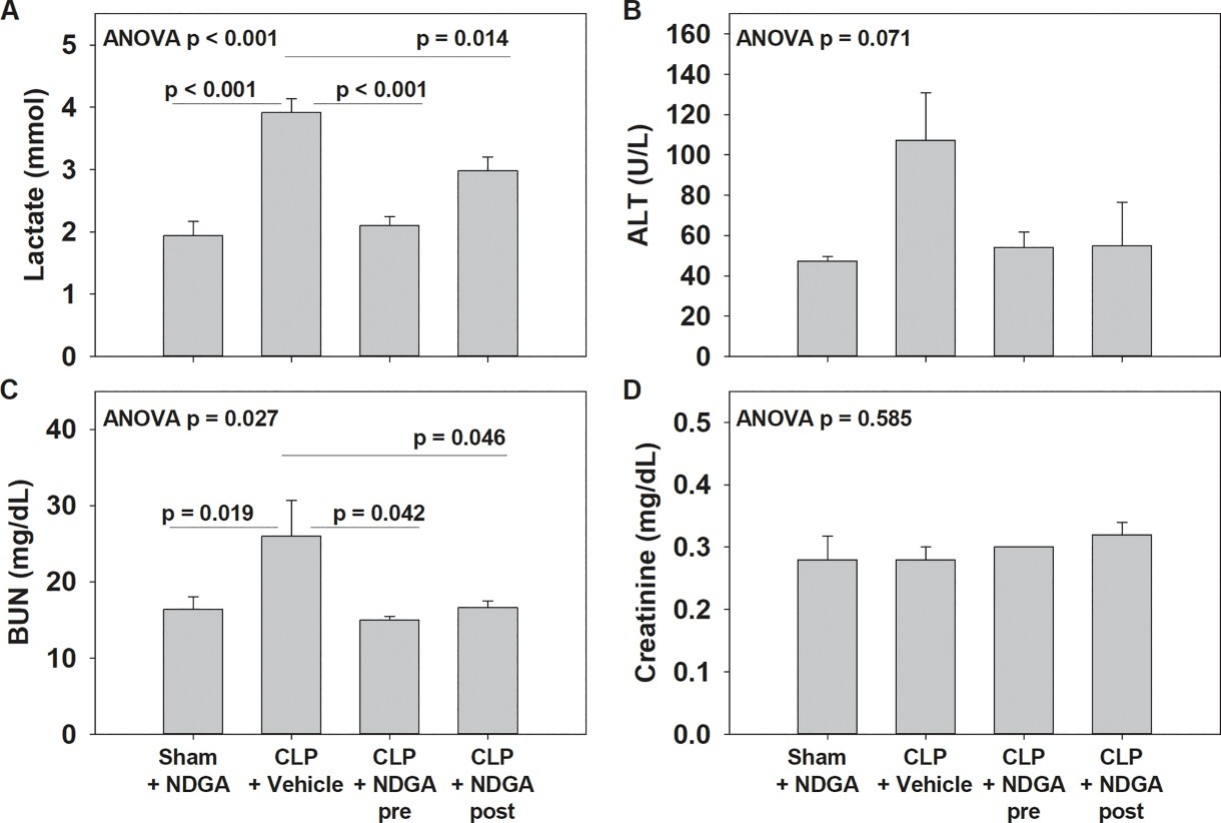
Quantification of metabolic status, liver injury, and kidney injury. (A) Quantification of tissue elevations in lactate to quantify derangements in acid base status and sepsis severity (n = 5 in each group). Significant reduction in serum lactate were seen among groups given NDGA both before and 6h after CLP when compared with CLP animals; (B) Quantification of ALT as a marker of liver injury. No differences were seen between groups; (C,D) Quantification of serum BUN and Cr as measures in kidney injury. Among BUN, significant reductions were seen among the sham group and groups given NDGA both before and 6h after CLP when compared with CLP animals. No differences were seen among Cr between the groups.

Lactate was elevated among CLP-vehicle animals and was significantly higher than sham surgery animals (1.9, p<0.001, Fig 4A). Both pre- (p < 0.001) and post-treatment (p = 0.014) with NDGA led to significantly lower levels of lactate compared with the CLP-vehicle group.

### Liver and kidney injury

There were no differences between the groups when comparing ALT as a marker of liver injury (Fig 4B). BUN was significantly higher in the CLP + vehicle group (26.4) compared with the sham group (16.4, p = 0.019), as well as the CLP + NDGA-pre group (15.0, p = 0.042) and the CLP + NDGA = post group (16.6, p = 0.046, Fig 4C). There were no differences between the groups seen with regards to creatinine (Fig 4D).

### Survival

**Fig 5:**
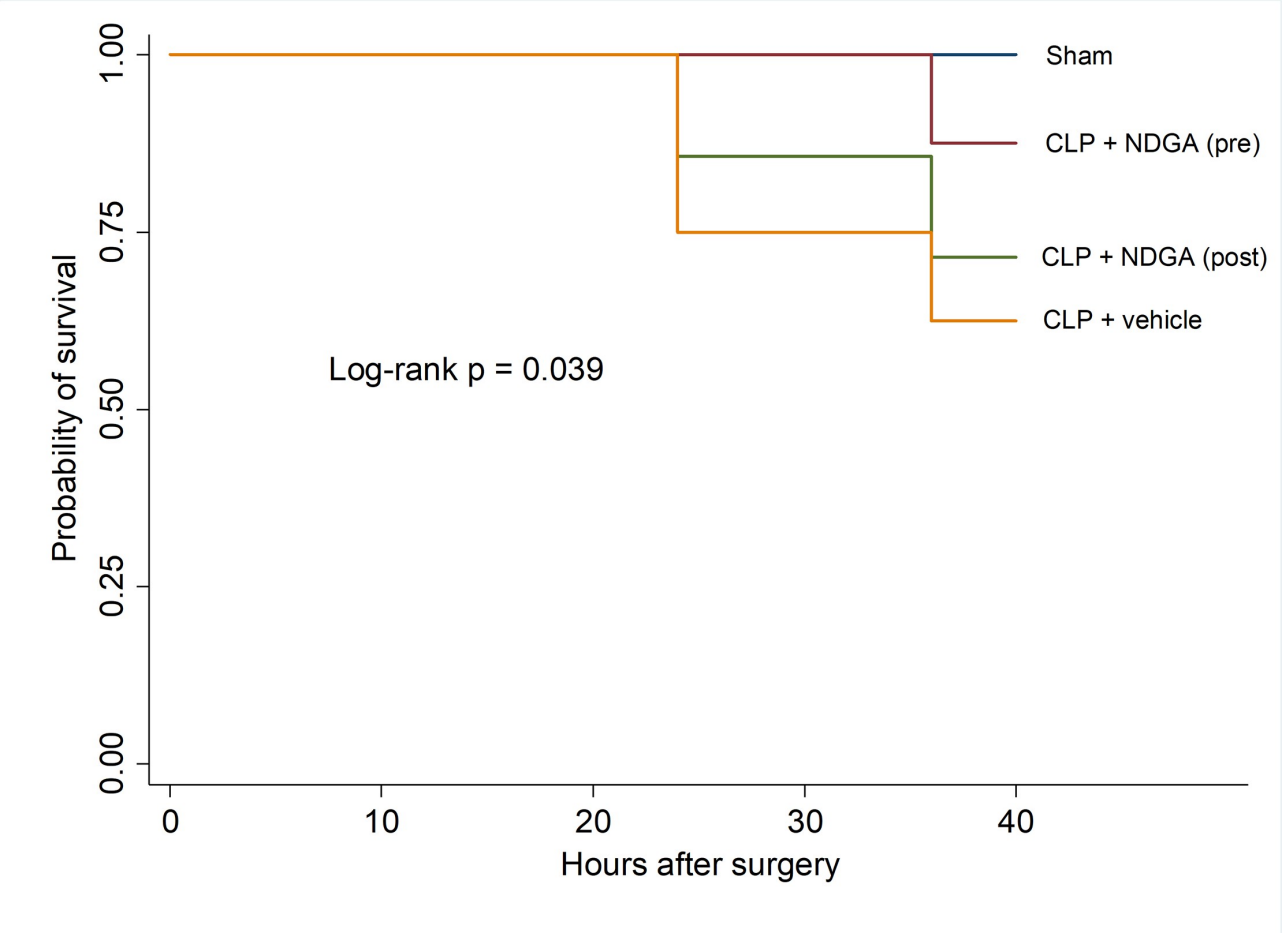
36 hour survival rate among all groups. A significant improvement in mortality is seen when a single dose of NDGA is given either at the time of induction of CLP or 6 hours after induction of CLP (n = 8 in each group).

One hundred percent (8/8) of the sham surgery animals survived the entire 36-hour experimental period. In septic animals without NDGA treatment, the survival rate at 36 hours was 62.5% (5 out of 8, 2 animals euthanized early for moribund status). In the CLP-NDGA pre- and post-sepsis treatment groups, the survival rate at 36 hours were 87.5% (7 out of 8, 1 animal euthanized for moribund status) and 75% (6 out of 8, 1 animal euthanized for moribund status), respectively (Fig 5).

### Inflammation–IL-1b

The levels of inflammation were higher in all 3 groups than in the sham group (p<0.05), but there were no significant differences noted between the CLP-vehicle group, CLP-NDGA pre-treatment or CLP-NDGA post-treatment groups (p>0.05, data not shown).

## Discussion

Our data shows that in a CLP-induced rat model of sepsis, NDGA given at the time of sepsis initiation has a modest mortality benefit and ameliorates the development of lung injury as measured by pulmonary edema, oxygenation, lung histology, kidney injury as measured by blood urea nitrogen, metabolic stress as measured by lactate. Additionally, NDGA given even 6 hours after induction of sepsis brought about improvements in lung, kidney, and metabolic injury, suggesting potential clinical utility as an agent that may help to decrease secondary organ injury in the setting of sepsis.

Sepsis is intrinsically an issue of dysregulated hyperinflammation leading to multiple organ dysfunction. Despite multiple trials looking at the utilization of pharmacologic approaches to reducing sepsis-induced secondary organ injury such as acute respiratory distress syndrome (ARDS) and acute kidney injury, there is either conflicting evidence as in the case of glucocorticoids [20] or no evidence of benefit in the cases of ketoconazole [21], activated protein C [22], and n-acetylcysteine [23,24]. Thus, there is interest and need for testing of potential therapeutics in pre-clinical models. Drugs with pleiotropic mechanisms of actions, such as NDGA, are worth investigating as they act on multiple potential targets implicated in sepsis. The amelioration of multiple metrics of organ injury with NDGA post-treatment supports further investigation of this molecule.

While many drugs such as glucocorticoids work by attempting to downregulate the immune response, there is likely enough immune molecule redundancy that has led to inconsistent success in animal models and clinical trials. Interestingly, although NDGA has been shown to reduce IL-1 in other models, our data did not show any clinical reduction in this arm of the inflammatory cascade. This suggests that the molecules may have acted more through its actions as an anti-oxidant than an anti-inflammatory. In our untreated CLP animals, the clinical combination of an elevated lactate with no substantial elevation in serum creatinine or serum alanine aminotransferase suggests the lactate elevation did not occur from substantial tissue hypoperfusion or tissue hypoxia, but rather from bioenergetic dysfunction at the level of the mitochondria, which has been shown to be common in the setting of septic shock and correlated with outcomes [25,26]. Reactive oxygen species have been shown to have these effects on a functional level due to widespread dysfunction of mitochondrial oxygen utilization and vascular endothelial dysfunction, leading to impaired oxygen utilization, microvascular tone disruption and secondary organ injury. In addition, in the lungs, there is impairment in ion transport [27], and impaired surfactant production [28]. When given in animal models of ARDS, free radical scavengers have been shown to ameliorate lung injury [29–32], which seems consistent with our results.

Importantly, we have also shown that the drug ameliorates lung and metabolic injury even after the initiation of sepsis, which has been a limitation of drugs studied in the past. With regards to renal injury, we also saw an improvement in blood urea nitrogen with both pre- and post-injury treatment with NDGA compared with vehicle only groups, but no differences in creatinine among groups. This is likely due to the fact that isoflurane has been shown to ameliorate renal injury in the setting of CLP [33]. Although there was only a modest improvement in survival, particularly in the group given NDGA after CLP, it may be that further work assessing the dose response and more detailed mechanistic evaluation may further illuminate a more optimal dose and time of delivery.

Additionally, NDGA has a low toxicity profile at the doses employed, as sham animals given NDGA had no mortality and no evidence of organ injury in the organs that were measured. There have been rare reports of idiosyncratic liver injury from chaparral–NDGA’s parent plant–in the past [34], but we did not see any elevation in serum transaminases in any of the groups given NDGA. Although not tested by our lung injury model, ARDS also occurs secondary to direct lung injury from both viral and bacterial infections. In an in vitro model, NDGA has been shown to have anti-viral effects against influenza A virus [35], possibly adding an additional layer of benefit in ARDS caused by direct viral lung injury.

We chose to use a model of non-pulmonary sepsis to best mimic common pathophysiologic changes seen in humans with sepsis and secondary organ dysfunction in the setting of an ongoing bacterial infection, but there are limitations to this model as well as our data. Although CLP represents a reproducible form of sepsis, the actual secondary organ injuries such as lung injury as assessed by hyaline membrane formation, inflammatory cell entry, and hypoxemia is relatively mild when compared with some other animal models of ARDS. Additionally, the CLP procedure itself requires the use of anesthesia, with many of the common anesthetics employed altering the very immune response under study and likely some organ injuries, as shown in our discordance between BUN and Cr with regards to kidney injury. CLP also fails to capture the complex interplay between coagulation and fibrinolytic systems that are seen in human sepsis. Additionally, there are distinct differences between the inflammatory mediators released from neutrophils in rats and humans. While NDGA has many possible routes of action, our data shows only improvement in end organ dysfunction and not the precise mechanism by which this improvement occurs. Although we speculate based on our organ injury data that it is a metabolic improvement from reduced oxidative stress, there are other avenues of anti-inflammation that could also be responsible for clinical improvement. Finally, we chose a model without antibiotics to induce more severe organ dysfunction. All of these differences potentially relate to the difficulty in translating therapeutic animal models to the clinical realm.

In this study, we showed that NDGA, a plant compound that has been shown to have anti-oxidant and anti-inflammatory properties, improves the degree of sepsis-induced lung and secondary organ injury in rats when given both at the time of as well as after CLP. Future studies will be required to characterize the mechanisms by which this is occurring, to assess response at different doses, to assess efficacy in different injury models in different species, and to determine whether these changes would translate to the same disease process in humans.

## Supporting information

S1 Data set (XLSX)
